# Using the Possibilities of Russian Space Medicine for Terrestrial Healthcare

**DOI:** 10.3389/fphys.2022.921487

**Published:** 2022-05-13

**Authors:** Oleg I. Orlov, Mark S. Belakovskiy, Anna R. Kussmaul, Elena S. Tomilovskaya

**Affiliations:** Institute of Biomedical Problems of the Russian Academy of Sciences, Moscow, Russia

**Keywords:** space medicine and biology, countermeasures, functional state assessment, kinetosis, telemedicine, small businesses

## Introduction

During the operation of orbital stations, the methods and means of medical support for cosmonauts and astronauts, monitoring their health status are being constantly improved, knowledge about the capabilities of the person himself, about the methods for managing the processes of human body adaptation to changing and often harsh environmental conditions, is increasing. It is well known that the consequences of the impact of various spaceflight factors are also encountered to some extent in terrestrial life—hypodynamia, hypokinesia, increased background radiation, deafferentation, isolation, etc. That is why the current level of development of biomedical research makes it possible to use the results obtained to keep the health of people not only in space, but also on Earth (Space Physiology … , 2016; Grigoriev, 2007). Dietrich et al. showed in their review that space technology has an impact on many domains of activity on earth, including in the field of global health. Various health research and technologies developed for inhabited space flights have been adapted for terrestrial use (Dietrich et al., 2018).

The Institute of Biomedical Problems of the Russian Academy of Sciences (IBMP RAS)—is the leading institution in Russia in the field of space biology and medicine. It is responsible for medical and sanitary and hygienic support for the crews, as well as the creation of scientific equipment for solving the problems of medical support and the implementation of the Russian national program of biomedical research and experiments on the Russian segment of the International Space Station (ISS). In addition, the IBMP RAS conducts interdisciplinary fundamental and pilot research in the field of medical sciences, radiobiology, engineering science, biotechnology, etc.

The Institute has carried out significant scientific and applied research, obtained unique results, and developed modern equipment (Belakovskiy and Samarin, 2002, 2011; Space Medicine … , 2014). It is obvious that the main area of the research results implementation is the improvement of medical support for the health and performance of cosmonauts in-flight and after returning to Earth, but nevertheless, a significant part of them has practical importance for implementation in healthcare(Orlov et al., 2014; Orlov et al., 2021).

## Directions for Using the Achievements of Space Medicine

In terms of using the possibilities of space medicine and its achievements to ensure the health and treatment of people, there are several areas in which a targeted search is underway:

1) Extension and deepening of knowledge about human health.
2) The use of equipment and research methods used on board space-based platforms in healthcare practice, i.e. adaptation of means, methods, equipment and devices created to solve the problems of space medicine to the tasks of earth medicine.
3) Application of space technologies in medical practice.

The tasks of space medicine include managing the functions of the human body in extreme environmental conditions to ensure a high level of its performance and the obligatory maintenance of an optimal state of health. This research area has generated qualitative shifts in the approaches and methodology of modern medicine. The main idea is that practically for the first time a healthy person became the object of study for a doctor. Multilateral systematic examinations, the most detailed study of all life processes occurring in a healthy human body, have enriched medicine with knowledge of normal reactions to various environmental influences. Maximum consideration of the body’s reserves, an individual approach, the use of the most modern methods of medical science for remote monitoring and predicting the state of health, the search for the border between adaptive and pathological changes under the influence of extreme environmental factors, and the prevention of the consequences of these factors distinguish precisely the space branch of medicine. All this allows to better know the normal human physiology. It can be argued that the knowledge of “terrestrial” physicians about the mechanisms of a person’s spatial orientation, the vestibular apparatus, its structure and function, information about biomechanics, metabolism, cardiovascular and nervous systems was replenished due to the cosmonautics.

The most famous examples of such “enrichment” of knowledge are the problems of hypokinesia and “motion sickness” (kinetosis). Hypokinesia was one of the effective ground models for simulating the physiological effects of weightlessness. By now, many studies have been carried out, including those with the length of stay of a healthy person on bed rest up to one year. In these studies, not only many data were obtained on the state and functions of all systems of the human body under these conditions, but also various methods and means of correcting the ongoing changes, as well as complex rehabilitative measures, were tested. All this, together with the available clinical material, provide a complete picture of the pros and cons of keeping patients in bed for a long time. Kinetoses, which are a very acute problem of cosmonautics, have prevented many people from traveling freely using air, sea, and even road transport. Pharmacological agents developed for cosmonauts for the prevention and reduction of manifestations of the space form of kinetosis show their effectiveness in other forms of motion sickness. A complete solution of this problem will be a serious contribution not only to cosmonautics, but also to practical public health. (Grigoriev et al., 2001a; Grigoriev et al., 2001b; Grigoriev et al., 2002; Grigoriev et al., 2003; Grigoriev et al., 2004; Grigoriev et al., 2005a; Grigoriev et al., 2005b; Vinogradova et al., 2005; Grigoriev et al., 2008; Grigoriev et al., 2009; Kozlovskaya et al., 2009; Grigoriev et al., 2012; Shenkman et al., 2014; Kozlovskaya et al., 2019).

The results of studying the functions and performance of humans in space under extreme environmental conditions have deepened our knowledge of the individual characteristics of healthy people adaptation, of determining the criteria for normal and pathological reactions of humans in these conditions, as well as the first symptoms of pre-illness. The accumulated experience is used to study the contingents of people whose professional activities take place in extreme conditions. A comprehensive assessment of indicators characterizing the functional state of the body, in particular its adaptive capabilities, makes it possible to identify the body’s response to the environmental situation not after 4–5 years, when diseases occur under the influence of adverse environmental conditions, but in the first months, as soon as the primary reaction of the organism develops as a general adaptation syndrome. (Baevskiy, 1979; Baevskiy et al., 2002; Baevskiy et al., 2008; Orlov et al., 2013; Baevskiy et al., 2014).

Space medicine has developed and implemented in healthcare new criteria and standards for human tolerance to functional loads, such as dosed physical activity on a veloergometer, passive orthostatic and antiorthostatic tests, a test with negative pressure on the lower half of the body.

Finally, valuable experience was gained in spaceflights in solving social and psychological issues of ensuring the professional activities of small groups of operators in extreme conditions of isolation and stress, optimizing their interaction, work regime, and organizing psychological support measures. All this is directly related to the problems of “terrestrial” sociology.

Biological and medical devices designed for human spaceflights have some advantages over existing “ground” devices: portability, low weight, easy use, resistance to overload, shock, vibration, and temperature changes. These devices are competitive in the class of equipment for the organization of a medical service that provides emergency assistance directly in sites of natural disasters, catastrophes, for medical examination of the population in hard-to-reach areas, for underwater, aviation and marine medicine, as well as for the examination of athletes (Grigoriev et al., 2003; Baevskiy et al., 2008; Netreba et al., 2009a; Netreba et al., 2009b).

An important direction in the application of the unique experience of space medicine for the benefit of humanity is the widespread use of telemedicine (Telematics Systems … 1992; Krupinski et al., 2002; Wilhite et al., 2022). Many years ago telemetry facilities were successfully developed and used in space medicine. The main purpose of these systems was medical control in order to recognize deviations dangerous to the health of cosmonauts. Even now it is difficult to imagine a more remote access of a doctor to a “patient”, although quite healthy. Of course, modern computer technologies have greatly modified the possibilities of telemedicine access on board the space station, but they are now being used more successfully in real-time medical teleconferencing and delayed consultations.

In almost all countries, there is a real need for reliable communication systems capable of transmitting medical information. Russia is one of the countries that, due to geographical features and a number of other factors, needs most to introduce such systems and technologies into the practice of medicine and healthcare (Kamaev et al., 2001; Clinical telemedicine … , 2001). Consulting patients from remote regions by specialized medical centers of the country, creating a more effective system of primary, postgraduate and continuing medical education, organizing and conducting coordinated research programs on the most actual problems, timely and targeted medical response to disasters and crises - this is an incomplete list of the main directions for application of telemedicine in clinical practice (Grigoriev et al., 2003; Perevedentsev and Orlov, 2012; Grigoriev and Sarkisyan, 1996).

## Platforms for Testing and Using the Developments of Space Medicine

The path from an idea to the implementation of a technology in clinical practice can take a long time and require a lot of resources. That is why one is interested in the mechanisms to speed up this process. An effective platform for testing technologies and adapting them for the clinic is ground-based analog studies. They allow such testing to be carried out in close interaction between science and technology, providing prompt feedback for developers, as well as working out organizational mechanisms for interaction between scientific organizations and industrial enterprises. The IBMP RAS has the capacity to conduct a wide range of analog studies (isolation, dry immersion, hypokinesia, short-arm centrifuge, etc.).

That is why it was invited to join the consortium, on the basis of which the World-Class Scientific Center (WCSC)—Pavlov Center “Integrative Physiology for Medicine, High-Tech Healthcare and Stress Resistance Technologies” was created. WCSCs are consortiums created within the framework of the Russian national project “Science” on the basis of an open competitive selection for grants in the form of subsidies from the federal budget. They were created in priority areas of scientific and technological development. The Pavlov Center included the Institute of Physiology named after I.P. Pavlov, Institute of Biochemistry RAS (as Coordinator), IBMP RAS, Institute of Evolutionary Physiology and Biochemistry named after I.M. Sechenov, St. Petersburg State Electrotechnical University “LETI” named after. V.I.Ulyanov (Lenin). Each element of the consortium has its own role. Thus, the Center for the Study and Prevention of the Effects of Long-Term Isolation was established at the IBMP RAS. Its purpose is to study the problems of stress caused by long-term physical and social isolation on the basis of model experiments and develop approaches to their prevention. These studies will allow substantiating the methods of psychological support, as well as suggesting new methods and modes of electromyostimulation and gravitational therapy for clinical practice. Thus, WCSC can serve as a successful example of mechanisms for adapting space technologies for the clinic.

However, further production, market launch and sales of products using registered intellectual property require a significant investment of resources, the availability of specialized competencies, as well as work in the appropriate legal field. Several mechanisms can be used to implement these processes. These include the following:

1) Providing advisory and expert support to small and medium-sized companies existing in the market,
2) Creation of small enterprises and conclusion of licensing agreements for granting non-exclusive licenses for the use of inventions (this is how several license agreements were concluded between the IBMP RAS and Center for Aerospace Medicine LLC, TsAM LLC) or the conclusion of agreements with already existing companies,
3) Creation of small innovative enterprises with the transfer of the right to use the results of intellectual activity (for example, contributions to the authorized capital) (this is how the Innovation Center of Space Medicine LLC was created).

Each of these mechanisms contribute not only to the expansion of IBMP’s opportunities for technology transfer, but also allow choosing the best way for such a transfer.

## Discussion

The application of methodological and technical developments of space medicine opens up unique opportunities for practical healthcare, changing the approaches to the structure of the medical care organization system. Space physiology and medicine provides rich material for creating methods, devices, technologies and knowledge that have the potential to improve the system of organizing medical care for the population of our country, and develop international cooperation in the field of practical healthcare. At the same time, the existing system for obtaining and applying scientific knowledge was initially not adapted to the large-scale implementation of research results into practice. In the process of its development, several working mechanisms of varying degrees of prevalence and effectiveness were worked out. The current mechanisms for testing and creating new technologies for healthcare based on existing space developments (research within the framework of the WCSC, the creation of SIB, etc.) make it possible to test, modify and find application for scientific developments, in particular, developments in space biology and medicine. The choice of a mechanism is influenced by a whole range of factors - from the prospects of development and the amount of necessary costs for the creation and implementation of a method or technology to the organizational and legal form of a scientific organization and the involvement of researchers and developers in the process of transferring technology into practice. In any case, the mechanisms require constant improvement, development and adaptation to the changing conditions of the economic environment. In order to make innovative work in this area more meaningful, effective and dynamic, it is necessary to form a social order, as well as more actively involve non-state structures that could suggest the most promising directions of the commercialization projects, from their point of view. This is what will allow the introduction of the developed “flight” technologies into clinical practice and biotechnological area to remain a significant component of medicine in the 21st century.
